# A translationally informed approach to vital signs for psychiatry: a preliminary proof of concept

**DOI:** 10.1038/s44277-024-00015-8

**Published:** 2024-08-26

**Authors:** Meredith L. Wallace, Ellen Frank, Colleen A. McClung, Sarah E. Cote, Jeremy Kendrick, Skylar Payne, Kimberly Frost-Pineda, Jeremy Leach, Mark J. Matthews, Tanzeem Choudhury, David J. Kupfer

**Affiliations:** 1 Departments of Psychiatry, Statistics and Biostatistics, University of Pittsburgh, Pittsburgh, PA USA; 2 Departments of Psychiatry and Psychology, University of Pittsburgh School of Medicine, Pittsburgh, PA USA; 3 Health Rhythms Inc., Long Island City, NY USA; 4 Department of Psychiatry, University of Pittsburgh School of Medicine, Pittsburgh, PA USA; 5 Ferkauf Graduate School of Psychology, Yeshiva University, New York, NY USA; 6 Department of Psychiatry, University of Utah, Salt Lake City, UT USA; 7 School of Computer Science, University College, Dublin, Ireland; 8 Department of Computing and Information Science, Cornell Tech, New York, NY USA

**Keywords:** Signs and symptoms, Human behaviour

## Abstract

The nature of data obtainable from the commercial smartphone – bolstered by a translational model emphasizing the impact of social and physical zeitgebers on circadian rhythms and mood – offers the possibility of scalable and objective vital signs for major depression. Our objective was to explore associations between passively sensed behavioral smartphone data and repeatedly measured depressive symptoms to suggest which features could eventually lead towards vital signs for depression. We collected continuous behavioral data and bi-weekly depressive symptoms (PHQ-8) from 131 psychiatric outpatients with a lifetime DSM-5 diagnosis of depression and/or anxiety over a 16-week period. Using linear mixed-effects models, we related depressive symptoms to concurrent passively sensed behavioral summary features (mean and variability of sleep, activity, and social engagement metrics), considering both between- and within-person associations. Individuals with more variable wake-up times across the study reported higher depressive symptoms relative to individuals with less variable wake-up times (B [95% CI] = 1.53 [0.13, 2.93]). On a given week, having a lower step count (−0.16 [−0.32, −0.01]), slower walking rate (−1.46 [−2.60, −0.32]), lower normalized location entropy (−3.01 [−5.51, −0.52]), more time at home (0.05 [0.00, 0.10]), and lower distances traveled (−0.97 [−1.72, −0.22]), relative to one’s own typical levels, were each associated with higher depressive symptoms. With replication in larger samples and a clear understanding of how these components are best combined, a behavioral composite measure of depression could potentially offer the kinds of vital signs for psychiatric medicine that have proven invaluable to assessment and decision-making in physical medicine. Clinical Trials Registration: The data that form the basis of this report were collected as part of clinical trial number NCT03152864.

## Lay summary

In this study, we linked passively sensed behavioral smartphone data with repeatedly measured depressive symptoms to evaluate which features could lead towards a behavioral vital sign for depression. Higher depressive symptoms were found among those having more variable wake-up times, lower step count, slower walking rate, more time at home, and lower distances traveled. Future studies could help us understand how these measures are best combined as a vital sign for depression.

## Introduction

Vital signs have been useful in the assessment of physiological health for centuries. Traditionally, these standardized measures included pulse, respiratory rate, temperature, and blood pressure. Oxygen saturation, pain, and smoking status have also become integrated into healthcare evaluations in the past few decades [1–3]. More recently, clinicians and researchers have proposed vital signs for mental health, including feeling “completely overwhelmed” among dementia caregivers [4], physical activity among people with schizophrenia [5], wellbeing among people with diabetes [6], and distress among cancer patients [7]. Although many integrative healthcare systems recognize the value of tracking vital signs for mental health [8], the challenge has been to find vital signs that are scalable, objective, and reliable.

In our own search for vital signs for mental health, we consider the potential value of the social zeitgeber hypothesis [9, 10], which provides a translationally relevant conceptual model for understanding the biological mechanisms linking our behavior, interactions, and daily routines to our mood [11–14]. According to this model, challenges to the body’s circadian system are at the center of many of the disturbances (i.e., criterion symptoms) seen in mood disorders. The social zeitgeber hypothesis also highlights interactions with other individuals and social role demands as determinants of daily routines that serve to regulate mood. Regularity of daily routines (e.g., the timing of sleep, activity, meals, and interpersonal contact) support a healthy circadian system, whereas irregularity of these routines could challenge circadian rhythmicity, triggering new episodes of mood disorder or serving as a factor in maintaining the mood disturbance [9, 10]. Multiple studies have validated the social zeitgeber model with respect to a range of psychiatric disorders including bipolar disorder [15, 16], depression [17, 18], anxiety symptoms [19], alcohol use [20, 21], and severity of post-traumatic stress disorder symptoms [22].

In the decades since the initial promulgation of the social zeitgeber hypothesis, animal models have allowed us to further probe the mechanisms linking the circadian system to mood regulation. These models implicate the suprachiasmatic nucleus (SCN) of the hypothalamus as the master clock that regulates the synchronization of endogenous cellular activity and gene expression over the course of an approximately 24-h cycle [23–26]. Circadian genes are expressed throughout the body and brain, regulating multiple systems such as immune function, monoamine transmission, neurogenesis, and metabolism – all of which can disrupt mood [27]. There is now substantial evidence that circadian genes control the rhythmicity of neuromodulators critical for mood, including serotonin, norepinephrine, and dopamine, which also exhibit circadian rhythmicity in their synthesis, release, and reuptake [25, 28–31]. Finally, in some animal models, external factors including changes in behavior and social interactions have been shown to alter the rhythmicity and synchronicity of endogenous circadian cycles and, in turn, impact mood or proxies thereof [32].

While animal research facilitates continuous and invasive monitoring of sleep/wake behavior and locomotor activity, human research conducted in the individual’s natural environment has been understandably limited. The recent availability of commercial smartphones may offer a transformative approach to measurement in psychiatric medicine, providing continuous, objective, real-world assessments of functioning. Methodological advances allowing for computationally efficient machine learning models that integrate high-dimensional features across multiple data streams have further expanded ways in which we can use smartphone data to understand how human behavior and interactions may be linked to mood. Numerous studies have now applied machine learning to smartphone data to measure or predict symptom severity in the context of depression [33] and bipolar disorder [34], with some studies showing relatively good performance [34].

Despite the recent wave of investigations into digital phenotyping, our field has not yet been able to identify the specific features – or combinations of features – that have the greatest potential to contribute to vital signs for depression. This may be a result of gaps in the digital phenotyping literature, combined with limitations of some current studies. Specifically, many digital phenotyping studies have had relatively large amounts of missing data, fail to instruct participants to keep the phone on or near their person during waking hours, are restricted to specific operating systems (e.g., allowing only Android and not iOS phones), and lack simultaneous active and passive data collection [35]. Furthermore, studies may employ machine learning methods that are challenging to interpret in a clinically meaningful context or lack conceptual models with a strong translational basis.

To begin to address these limitations, we developed Cue (Health Rhythms, Inc., New York, NY, USA), a smartphone-based platform for the continuous, objective measurement of mood-relevant behaviors and rhythms on both iOS and Android devices. With this technology, we can extract numerous behavioral inferences with potential relevance to depression and to mental health more broadly. The behavioral inferences drawn from the Cue platform naturally fell into four categories based on the social zeitgeber model**:** proxies for sleep, activity, and social interaction, as well as the rhythmicity of features within each of these three categories. Motivated by translational studies [36, 37], we aimed to identify which behavioral inferences are useful for indicating *who* is at risk overall, as well as *when* a person is at risk of increased depression severity.

Sleep characteristics related to timing, duration, continuity, and regularity are central components of ‘sleep health’ [38] and are thought to have bi-directional associations with depression [39]. Wake-up time directly ties to exposure to morning light, a key zeitgeber [40], and initiates a cascade of signals associated with the waking versus sleeping state [41–43]. We hypothesized that more variable wake time, longer time in bed, and greater sleep interruptions would be associated with increased depressive symptoms.

Features reflecting inactivity and psychomotor slowing or agitation are criterion symptoms of depression, as well as a variety of other mental disorders. Step count represents an objective measure of a patient’s level of activity, while walking rate can reflect psychomotor slowing or agitation. With respect to depression, we hypothesized that decreased step count and walking rate would be associated with increased depressive symptoms.

Given the importance of interpersonal interactions as social zeitgebers (i.e., determinants of a person’s routine), passively sensed proxies for levels and timing of social engagement may be important behavioral vital signs for depression. Of particular interest, Mohr and colleagues suggested that ‘location entropy’ – which quantifies the uniformity of time spent at the various locations to which one goes – could represent a proxy for social interactions that serve as anchors to an individual’s social routines [44, 45]. We expected that less social engagement – indexed by lower location entropy, more time at home, and lower distances traveled – would be associated with higher depressive symptoms.

In the current study, we report on an initial effort to explore passively sensed behavioral features – focusing on 24-h rhythms of sleep, activity, and social engagement factors – that could eventually contribute to a clinically meaningful vital sign for depression.

## Methods

### Study population and design

The study sample consists of adult outpatients originally recruited for a randomized clinical trial (RCT), for which the sample, methods, and results have been described previously [46]. Briefly, outpatients in treatment at the University of Utah Department of Psychiatry who previously indicated willingness to participate in research were contacted consecutively by phone to inquire about their interest in participating in a study of a digital intervention platform for depression. All procedures involving human subjects/patients were approved by the University of Utah IRB (approval #00098927) and all participants gave written informed consent. The RCT participants could be at any point in their treatment course, and most had relatively low levels of depression severity as assessed by the PHQ-8 [47] at the outset of the trial. Participants provided continuous behavioral data over the 16-week study period and completed the PHQ-8 approximately every two weeks. Because the fully virtual nature of the trial precluded rapid response to the endorsement of suicidality, the ninth item of the full PHQ-9, suicidality, was not assessed. Participants were instructed to keep the phone on or near their person during waking hours.

The original 16-week trial enrolled 135 participants. To be included in the present report we further required that a participant have sufficient passive sensing data to ensure reliable measurement, defined as ≥5 days of passive sensing in the week prior to a PHQ-8 report. One hundred and thirty-one participants met this requirement, and their available data are used in the present study. Of these 131 participants, 119 (91%) completed the trial, 4 (3%) were terminated per protocol because of a change in their psychiatric treatment, and 8 (6%) were lost to follow-up.

### Measures

#### Outcome

Our outcome is depression severity as measured by the PHQ-8, an established measure of depression severity [47] that excludes the ninth (suicidality) item of the original PHQ-9 [48]. In the RCT, PHQ-8 was measured at baseline (week 0) and roughly every two weeks thereafter through week 16 (i.e., nine times total). For this analysis, we omit the baseline PHQ-8 because there was not sufficient passive sensing data measured prior to baseline. Thus, we consider up to eight repeated PHQ-8 observations measured between weeks two and 16.

#### Passive sensing features

Our pipeline automatically extracts numerous daily features (i.e., behavioral inferences) that are potentially relevant to mental health from four passively sensed data streams (location, pedometer, activity, and display status). Informed by the literature and our clinical hypotheses and expertise, we selected an initial subset of 22 daily features that may inform the development of a behavioral vital sign for depression. These features address aspects of sleep/wake regulation, rest/activity patterns, and proxies for social engagement.

For each of the 22 daily features, we computed a series of ‘*weekly passive sensing summary features*’ corresponding to each repeated PHQ-8 observation. Specifically, for a PHQ-8 on a given day *t*, we computed the mean and standard deviation (SD) of the daily passive sensing feature from day *t-7* through the time of the PHQ-8 on day *t* (i.e., seven full days prior to the day of the PHQ-8 plus any time prior to the PHQ-8 on the day it is measured). For valid measurement, we required that at least five days of passive sensing data were available to compute each feature. For an individual *i*, we denote a weekly passive sensing summary feature for week *t* as ${X}_{{it}}$. This produced 44 types of weekly passive sensing summary features corresponding to each repeatedly measured PHQ-8 observation (22 means, 22 SDs).

For each participant *i* and each of the 44 types of weekly passive sensing summary features, we also computed a ‘*global passive sensing summary feature*’ as the mean of the weekly feature across the observed study period, denoted ${\bar{X}}_{i}$. It reflects the typical level of the summary feature across the entire duration of their study participation. We explored Spearman correlations among the 44 global passive sensing features. Informed by these correlations, we further selected 18 summary features that were relatively independent, and which had the most interpretability and face validity for the social zeitgeber model.

For sleep, we selected the means and SDs of our proxies for bedtime, wake-up time, and the time spent in bed (i.e., time between bedtime and wake-up time) during the week prior to each PHQ. We also selected the mean number of sleep interruptions. The SD was not considered because of its high Spearman correlation (*r* = 0.95) with the mean number of interruptions. Prior work indicates that features extracted from passive sensing can be reasonable proxies for these measures of sleep [44, 45].

For activity, we considered the means and SDs of the total daily step count (per 1000 steps) and the walking rate per second within periods of activity throughout the day.

For social engagement, we considered the mean normalized location entropy and the means and SDs of the number of unique locations visited, time at home, and distances traveled. The normalized location entropy ranges from 0 to 1, with higher values indicating a more equal distribution of time spent at each location visited. We considered only the mean and not the SD of location entropy because location entropy inherently incorporates a component of regularity. Distances traveled is a measure of the typical distances traveled from the user’s home to various locations on a given day in meters, weighted by the amount of time spent at each location. To meet regression assumptions regarding the normality of residuals, we log-transformed the number of location clusters and distances traveled prior to computing summary features.

For a given individual on a given calendar day, we computed the coverage of each of the four raw data streams (location, pedometer, activity, and display status) as the sum of the durations of stream events for that day divided by the total duration of the day (usually 24 h, with adjustments as needed for travel across time zones). Overall coverage for a particular individual and day was then computed as the mean of the four stream-specific coverages.

Descriptions and technical details of the 18 selected passive sensing summary features are provided in the Supplement.

#### Covariates

Sociodemographic and clinical features considered relevant to the social zeitgeber model and to depression in general included occupation (employed vs. not employed), living status (with others vs. alone), age, and gender. These features were selected as covariates based on prior work indicating these features have implications for depressive symptoms, sleep, activity, and social rhythms [10, 46, 49]. Additional covariates informed by the original RCT study design and analysis included the week of measurement (square root transformed to accommodate participants’ initially steeper decrease of depressive symptoms during the RCT), the treatment condition (experimental vs. control), and the treatment by week interaction. Our interest in the present report is not in the treatment effect; however, we include it here to control for any potential study design effects.

### Data analysis

For each participant *i*, we computed the ‘*weekly passive sensing feature deviation’* at week *t* as the difference between the weekly passive sensing summary feature at week *t* and the global passive sensing feature, $\triangle {X}_{{it}}={X}_{{it}}-{\bar{X}}_{i}.$ It reflects the extent to which a participant’s weekly summary feature corresponding to the week prior to *t* deviated from their own ‘global’ level across the study period. To investigate missing data, we computed and summarized the numbers of observed PHQ-8 scores and corresponding valid passive sensing features for each person, with a maximum of eight possible repeated measures between weeks 2 and 16.

For our primary analyses, we used linear mixed-effects models to simultaneously regress the repeatedly measured PHQ-8 score on the global feature ${\bar{X}}_{i}$ and the weekly feature deviation $\triangle {X}_{{it}}$. This approach facilitates the examination of both between-person associations (via global features) and within-person associations (via weekly feature deviations) [50]. Put another way, the consideration of both global features and weekly feature deviations allows us to simultaneously examine: (1) whether people with higher/lower global features tend to have higher/lower PHQ-8 scores on average, relative to other people (i.e., *who is at risk?*); and (2) whether having a deviation from one’s global feature average might be associated with higher/lower PHQ-8 scores for that week (i.e., *when is a person at risk?*). Models were fit for each set of passive sensing features separately and adjusted for covariates described above. Random intercept and slope terms were included to account for within-subject correlations. Finally, to quantify and compare the contributions of each passive sensing feature to explaining model variance, we extracted fit indices including the marginal *R*^2^ (the percent of model variance attributable to the passive sensing features and covariates) and the change in marginal *R*^2^ (Δ*R*^2^) attributed to the set of passive sensing features.

Mixed-effects modeling was performed using the nlme package [51, 52] in R Studio 22.07.0 [53] and model fit estimates (marginal *R*^2^, Δ*R*^2^) were computed using the R insight package [54, 55]. Because our analyses are exploratory – with the goal of generating hypotheses that will be tested in future confirmatory studies – we do not perform multiple comparison corrections, per current statistical recommendations [55, 56].

Details regarding power for this study are provided in the Supplement.

## Results

Table 1 describes the clinical and demographic characteristics of the 131 individuals included in this analysis. Descriptive summaries of the global passive sensing features are provided in the Supplement. The 18 features had a median Spearman correlation magnitude of *r* = 0.23 (Q1 = 0.13, Q3 = 0.33). Pairs of features with large associations (defined as *r* ≥ 0.70) were time at home and normalized location entropy (*r* = −0.73) and mean step count and mean walking rate (*r* = 0.86). Otherwise, features had small-to-moderate associations.

**Table 1:** Baseline sociodemographic and clinical characteristics (*N* = 131)

	Mean (SD) or *N* (%)
Clinical characteristics	
Age	32.8 (11.3); median 31
Gender	
Female	96 (73.3)
Male or Other	35 (26.7)
Occupational/School status	
Full time	78 (59.5)
None	19 (14.5)
Part-time	34 (26.0)
Living status	
Alone	22 (16.8)
Family	84 (64.1)
Unrelated others	25 (19.1)
Lifetime diagnosis	
Anxiety disorder only	15 (11.5)
Mood disorder only	24 (18.3)
Anxiety & mood disorder	92 (70.2)
Pharmacotherapy	102 (80.2)
Baseline PHQ-8	11.00 (4.75)

During the study period (weeks 2–16), participants had a mean (SD) of 7.5 (1.3) observed PHQ-8 measurements of a possible eight. They averaged >7 valid repeated measures for each weekly passive sensing summary feature, also out of a possible eight. Further, across both people and days within the 16-week study period, we observed a median of >99% coverage of the passive sensing features. Thus, study participants had relatively few missing data points in either the PHQ-8 outcome or the passive sensing features.

### Model results

#### Sleep

**Fig. 1:**
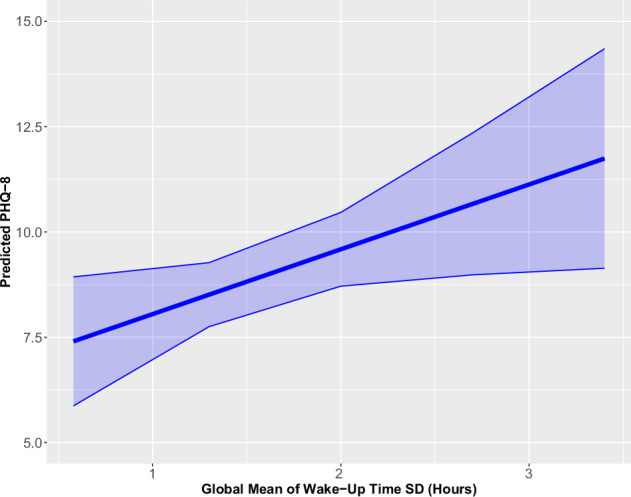
Predicted PHQ-8 (95% confidence interval) for the global mean of wake-up time standard deviation (SD).

Participants with higher global wake-up time variability (i.e., higher SDs of wake time over their study period) had significantly higher PHQ-8 scores relative to other participants (B [95% CI] = 1.53 [0.13, 2.93]; see Fig. 1). The Δ*R*^2^ attributed to wake-up time variability was 0.024. Other sleep features (mean wake-up time; mean sleep interruptions; means and SDs of sleep start and time in bed) were not associated with depressive symptom severity.

#### Activity

**Fig. 2:**
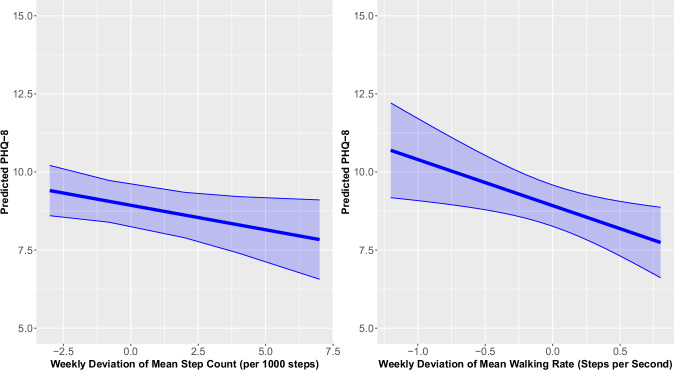
Predicted PHQ-8 (95% confidence interval) for the weekly deviation of mean step count (left) and weekly deviation of mean walking rate (right).

On weeks when participants had lower total step count per 1,000 steps and decreased walking rate per second, their PHQ-8 scores were significantly higher relative to their usual levels (Step Count B [95% CI] = −0.16 [−0.32, −0.01]; Walking Rate B [95% CI] = −1.46 [−2.60, −0.32]; see Fig. 2). The Δ*R*^2^ for step count and walking rate were 0.019 and 0.013, respectively. SDs of the step count and walking rate were not associated with depressive symptom severity.

#### Social engagement

**Fig. 3:**
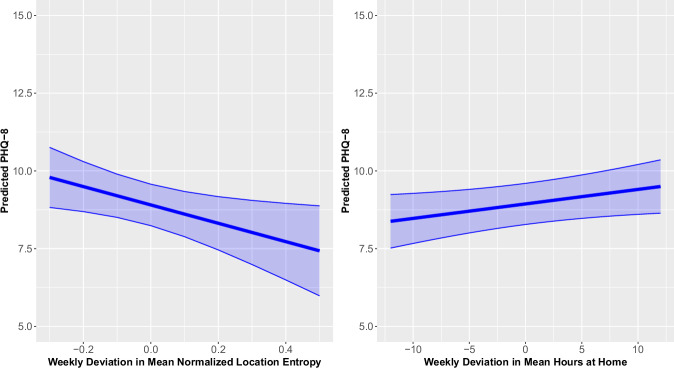
Predicted PHQ-8 (95% confidence interval) of the weekly deviation in mean normalized location entropy (left) and weekly deviation of mean hours at home (right).

On weeks when participants had lower normalized location entropy and greater hours at home, their PHQ-8 scores were significantly higher relative to their usual levels (Normalized Location Entropy B [95% CI] = −3.01 [−5.51, −0.52]; Time at Home B [95% CI] = 0.05 [0.00, 0.10]; Fig. 3). The Δ*R*^2^ for normalized location entropy and time at home were 0.005 and 0.009, respectively. Additionally, participants with higher mean levels of distances traveled across the study duration had significantly lower PHQ scores relative to other participants (B [95% CI] = −0.97 [−1.72, −0.22], Supplement), with Δ*R*^2^ = 0.033. SDs of time at home and distances traveled were not associated with depressive symptom severity. The mean and SD of the number of location clusters were also not associated with depressive symptom severity.

Full model results and fit indices are provided in the Supplement.

#### Illustrative examples of passive sensing features that could inform a vital sign for depression

**Fig. 4:**
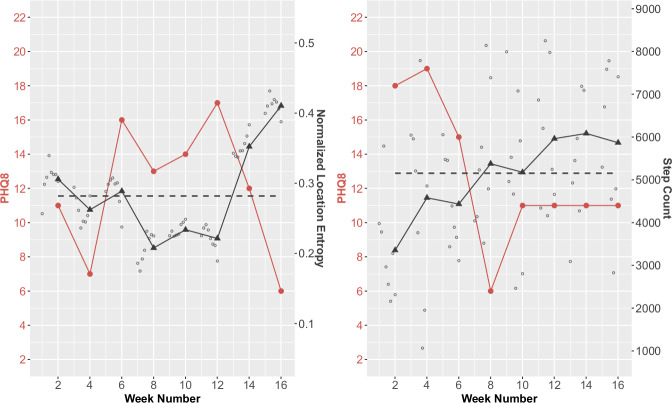
Study participant data showing how PHQ-8 scores track with normalized location entropy (left) and step count (right).

Figure 4 illustrates how passive sensing features could be used to understand *when* people may have higher symptom levels, considering two different features and two different participants. For normalized location entropy (Fig. 4, left), the weeks with lower PHQ scores generally have a location entropy that is greater than or similar to the participant’s typical location entropy, whereas weeks with a higher PHQ score generally have a location entropy that is lower than the participant’s typical location entropy. For step count (Fig. 4, right), the weeks with lower PHQ scores tend to have a step count that is higher than the participant’s typical step count, while the weeks with a higher PHQ score tend to have a step count that is lower than the participant’s typical step count.

The Supplement provides additional examples illustrating how passive sensing features could be used to understand *who* has higher symptom levels across the entire study period.

## Discussion

This study was developed from a translational social zeitgeber model that has been validated by decades of research and focuses on the importance of regularity of daily routines to circadian health and – through multiple pathways – to mood regulation. Guided by this translational model, we developed a digital platform for the continuous, objective monitoring of behaviors relevant to mental disorders, paired with regular subjective assessments. We explored how passive sensing proxies of sleep, activity, and social engagement relate to self-reported depression severity in a group of 131 psychiatric outpatients. Across findings and consistent with our conceptual model, we observed that the variability and deviations of these features – rather than the levels of the features themselves – were most related to depressive symptoms.

With respect to sleep, participants with greater variability in our passively sensed proxy for wake time had significantly higher PHQ-8 scores over the 16-week study period relative to participants with lower variability in wake time. Regular wake time promotes circadian rhythmicity through signals initiated by the act of arising from bed, such as signals from the SCN to the digestive and elimination systems to ready them for food digestion and urination [41–43]. Even if we do not arise from bed, the simple act of awakening and opening our eyes is one of the strongest and most important zeitgebers for establishing circadian rhythmicity [57]. An important caveat to this finding is that passive sensing is not expected to provide a precise measure of actual physiological awakening. Despite this limitation, the algorithm we use for wake time has direct relevance to the social zeitgeber model because it represents an initial post-sleep engagement with the world that can be relatively reliably estimated from smartphone sensor data. More research should be conducted to distinguish whether variability in the time of physiological awakening – versus variability in the time of post-sleep engagement with the world – is more strongly associated with depressive symptoms.

Our findings for activity are consistent with the inclusion of inactivity and psychomotor slowing as a criterion symptom of depression [39]. Regular activity guides the circadian clock to a 24-h schedule; for example, exercise can entrain circadian rhythms in mice independent of light exposure [58]. Regular activity also promotes thermogenesis, strong immune function, healthy hormone release, metabolism, reproduction, and stem cell development – all of which are plausible pathways connecting circadian health to mood regulation [59]. Activity levels during daylight also train the body to be awake during that time and promote consistency in zeitgeber cues [60]. In our study, weeks when step count and walking rate were lower than usual corresponded to weeks with higher depressive symptom severity. This is consistent with animal models where lower locomotor activity is used as one proxy for depression symptoms [61].

Passive sensing proxies for social engagement indicated which weeks and which people had higher depressive symptom severity. Weeks with lower normalized location entropy and more time at home than usual corresponded with higher depressive symptom severity within an individual. In contrast, people with lower distances traveled from home tended to report greater depressive symptom severity over the course of the study. Although light seems to be the most potent stimulator of the SCN, other stimuli such as food consumption, drugs, and social cues (or lack thereof) can trigger neurobiological systems that cause processes to lose synchronicity with the SCN and the rest of the body [62]. In their review, Mistlberger and Skene [63] found strong evidence that social influences can entrain mammalian circadian rhythms. In humans, Ronneberg et al. [64] and Foster et al. [65] demonstrated that social cues, new environments, mealtimes, and work schedules – along with light exposure – can influence circadian rhythms. Thus, social engagement and time spent outside the home support healthy circadian rhythms by regulating one’s schedule and by providing the social cues inherent in interpersonal interactions.

Although the sleep, activity, and social engagement features we identified are exploratory and must be further validated prior to their incorporation into clinical interventions, it is promising that they are actionable in much the same way as classic physical vital signs. For example, in clinical practice, patients who exhibit variable wake times could be encouraged to establish more regular sleep timing, either through their own effort, through participation in a course of behavioral psychotherapy such as CBT-I [66, 67], or a digital intervention such as Sleepio [68]. Similarly, activity could be useful for identifying which weeks a patient is experiencing higher-than-usual depressive symptom severity. Indeed, some of our own work with patients with bipolar disorder [69], as well as the work of others, suggests that even modest increases in relatively low-intensity exercise such as walking can have a positive impact on mood symptoms [70]. Social engagement is also something the clinician could try to address. Whether a clinician treating depression is working from simple common sense, a cognitive behavioral, or an interpersonal model, encouraging and planning for regular social engagement in an isolated patient is frequently an important part of the intervention [71–73].

Our study leverages a combination of clinical and ecological validity that is directly relevant to our conceptual model [74]. The use of personal devices instead of study-imposed devices (such as study-specific phones, watches or other wearables) and passive sensing data collection provided minimal disruption to participants’ environments and routines. This user-friendly and scalable approach resulted in high adherence to self-report data and very high sensor coverage. The concepts of behavioral vital signs provide a clinically interpretable, relevant, objective, digestible summary of a person’s psychological state that could inform treatment [75]. Moreover, our use of statistical models that incorporate both within-person (i.e., which weeks) and between-person (i.e., which people) vital signs advances our conceptualization of the types of features that could be informative.

Our study has limitations that are necessary to clarify and that point to future directions. First, this analysis is based on a relatively small sample observed in only a single site and is somewhat younger and less racially and ethnically diverse than is typical in some outpatient settings, which limits generalizability. Nonetheless, from a diagnostic perspective, the sample is mostly representative of the population being treated in outpatient psychiatric services, demonstrating a mix of lifetime mood, anxiety, and other disorders. Second, most participants in the study from which these data were drawn reported relatively mild depressive symptoms at study onset and there was limited variability in depressive symptom severity over the course of the study – perhaps because most participants were already taking antidepressant medication. These study features are hurdles to identifying strong associations; thus, we are encouraged by our findings suggesting that relatively small numerical differences, for example in number of steps per day, were nonetheless related to self-reported depression severity. Third, although we had clear hypothesis-driven constructs of interest (sleep, activity, social engagement, and regularity of routine) drawn from a validated conceptual model, ultimately ours is an exploratory study and the findings we report should be viewed cautiously until further replication.

Given these limitations, our findings must be replicated in a larger sample with a greater range of depression severity across follow-up and a clinician-evaluated measure of depression symptoms prior to generalization to other contexts. Such larger studies could also facilitate the examination of potential interactions of passive sensing features and other sociodemographic and clinical features, or the role of season (e.g., winter, spring, summer, fall), which can impact mood, behavior, and circadian biology [76]. We also emphasize that these findings should not be construed as causal. While we observed associations between passively sensed measures of behavioral routines and depressive symptom severity, additional work must be performed to determine whether attempts to increase the regularity of these behavioral routines could be used to improve depressive symptom severity and/or prevent depressive relapse. Data from animal work could play a role in designing such human experiments.

The sleep, activity, and social engagement features that we identified in this exploratory study provide an initial framework for larger studies focused on developing a composite behavioral vital sign for depression. Thus far we have only proposed components of a behavioral vital sign for depression and have not yet addressed how these should be combined, weighted, or scaled for widespread use [77]. We are encouraged by these early findings and look forward to examining how these passively sensed behaviors could yield a composite vital sign for depression and other psychiatric disorders in future work.

## Supplementary information

Supplemental file
