# Supplementary material for: A translationally informed approach to vital signs for psychiatry: a preliminary proof of concept
Source: NPP Digit Psychiatry Neurosci. 2024 Aug 26;2:14. doi: 10.1038/s44277-024-00015-8 (PMC11619764; doi:10.1038/s44277-024-00015-8)
Supplement: Supplementary file 1 — Supplemental file [file 44277_2024_15_MOESM1_ESM.docx]

**A TRANSLATIONALLY INFORMED APPROACH TO VITAL SIGNS FOR PSYCHIATRY: A PRELIMINARY PROOF OF CONCEPT**

Meredith L. Wallace, Ellen Frank, Colleen A. McClung, Sarah E. Cote, Jeremy Kendrick, Skylar Payne, Kimberly Frost-Pineda, Jeremy Leach, Mark J. Matthews, Tanzeem Choudhury, David J. Kupfer

**Supplemental Materials**

Contents

[**1.** **Calculation of passive sensing features** 2](#_Toc172782029)

[Sleep Features 2](#_Toc172782030)

[Activity Features 2](#_Toc172782031)

[Social Engagement Features 2](#_Toc172782032)

[**2.** **Passive Sensing Descriptive Statistics** 4](#_Toc172782033)

[**Table S1.**  Daily Passive Sensing Features 4](#_Toc172782034)

[**Table S2.** Mean (standard deviation [SD]) of numbers of weekly passive sensing features available (i.e., at least five days of data prior to a PHQ8 score) across the 16-week study period. The maximum possible number of weekly passive sensing features is 8. 5](#_Toc172782035)

[Table **S3.**  Global Passive Sensing Features. SD = Standard Deviation. 6](#_Toc172782036)

[**3.** **Power Calculations** 7](#_Toc172782037)

[**4.** **Model Results** 8](#_Toc172782038)

[**Table S4.**  Results for the global mean and deviation of each social engagement feature. Bolded text indicates feature was statistically significant. (SD=Standard Deviation) 8](#_Toc172782039)

[**Table S5.**  Results for the global mean and deviation of each activity feature. (SD=Standard Deviation) 9](#_Toc172782040)

[**Table S6.** Results for the global mean and deviation of each sleep feature. (SD = Standard Deviation) 9](#_Toc172782041)

[**Table S7**. Explained variance in linear mixed effects models. Bolded items had a statistically significant between- or within-person feature in the model. 10](#_Toc172782042)

[**Figure S1.** Predicted PHQ-8 (95% Confidence Interval) for the global mean of distances traveled. 11](#_Toc172782043)

[**5.** **Illustrative Examples of Passive Sensing Features that Could Inform a Vital Sign for Depression** 12](#_Toc172782044)

[**Figure S2**. Study participant data showing how PHQ-8 scores track with wake-up time standard deviation (SD) for two participants. 13](#_Toc172782045)

# **Calculation of passive sensing features**

## Sleep Features

To create proxies for *Bed Time* and *Wake-up Time*, we used an algorithm to label each five-minute epoch as active (i.e., unlocked and/or moving for any portion of the time) or idle. After applying a moving average, we identified the longest period of time during which the phone is idle. We integrated this activity information with additional information from a sleep diary where participants self-reported bed time and wake-up time to define the main sleep period. The start and end of that period are defined as Bed Time and Wake-up Time.

*Time in Bed* is computed as the duration of time between the proxy for sleep start and the proxy for sleep end.

*Sleep Interruptions* is computed as the number of times that the moving average signal rises above a set threshold.

## Activity Features

*Total Step Count* is defined as the total number of steps per day as recorded by the pedometer.

*Walking Rate* is defined as the number of steps per second during walking bouts.

## Social Engagement Features

For each person, the ‘center location’ is defined by the latitude/longitude at which the participant spends the most time for the duration of the study. This is their presumed home.

*Time Spent at Home* for a given day is computed as the hours spent within 100 meters of the participant’s ‘center’ location on that day.

*Number of Location Clusters* for a given day is computed as the total number of new location clusters visited over the past 14 days. A new location cluster is defined as spending 5 minutes at a location more than 500 meters away from a prior location. If there are fewer than 14 prior days with observed data, the maximum number of observed days is used. The minimum number of location clusters is 1, indicating the person stayed at home.

*Normalized Location Entropy* (NLE) is computed as $NLE=-\frac{\sum_{c=1}^{C} p_{c}\ln(p_{c})}{\ln(C)}$, where $p_{c}$ is the proportion of time that the user spent within location cluster *c* over the past 14 days and *C* is the total number of location clusters. A ‘location cluster’ is initially defined by a latitude/longitude pair at which the user spent a minimum of 5 minutes. The duration of time spent at any other location within 500 meters of the centroid of location cluster *c* is added to the total duration at location cluster *c*. In this situation, the cluster’s centroid is updated from its previous centroid (initialized by the first latitude/longitude pair visited in that cluster) to the mean of that centroid and the newly visited location. If a new location is visited for more than 5 minutes and is more than 500 meters from an existing centroid, this location initializes a new cluster. The normalized location entropy ranges from 0 (spending the prior 14-day period at home) to 1 (indicating an equal proportion of time was spent at each of the C location clusters).

*Distances Traveled* (DT) is computed as $DT=\sqrt{\frac{\sum_{c} w_{c}d_{c}^{2}}{\sum_{c} w_{c}}}$, where $d_{i}$is the distance of location *i* from the centroid of locations for that day and The numerator is a weighted sum of squared distances traveled to each location *c*, and $w_{i}$ is the duration of time spent at location *c*. Thus, DT represents the typical distance traveled to each location *c*, weighted by the amount of time spent at location *c*. A person who only spent time at home would have DT=0. DT increases as more locations are visited and more time is spent at each of those locations.

# **Passive Sensing Descriptive Statistics**

**Table S1** outlines the selected weekly passive sensing summary features. **Table S2** shows the mean (standard deviation [SD]) number of weekly summary features observed across the course of the study. The maximum number of possible weekly summary features is eight, corresponding to the eight possible PHQ-8 observations across the course of the study. **Table S3** summarizes descriptive statistics of the global passive sensing summary features.

## **Table S1.** Daily Passive Sensing Features

| **Daily Feature** | **Definition** | **Weekly Summary Features Considered** |
| --- | --- | --- |
| Sleep | |  |
| Bed Time | Start of the main sleep period (HH:MM). | Mean, Standard Deviation |
| Wake-up Time | End of main sleep period (HH:MM). | Mean, Standard Deviation |
| Time from Bed to Wake-Up | Duration of time between bed and wake-up time. | Mean, Standard Deviation |
| Sleep Interruptions | Number of times the signal rises above a set threshold during the main rest period (i.e., time between Bed and Wake-up). | Mean |
| Activity | |  |
| Total Step Count | Total number of steps / 1000. | Mean, Standard Deviation |
| Walking Rate | Steps per second during walking bouts. | Mean, Standard Deviation |
| Social Engagement | |  |
| Normalized Location Entropy | Equality of the proportion of time spent at each unique location visited in the prior two weeks, ranging from 0 (all time at home) to 1 (equal proportion of time spent at each location cluster). | Mean |
| Number of Unique Locations | Number of unique locations visited in the prior two weeks. | Mean, Standard Deviation |
| Time Spent at Home | Hours spent within 100 meters of the participant’s ‘center’ location (their presumed home). | Mean, Standard Deviation |
| Distance Traveled | The typical distance traveled in meters from the participant’s ‘center’ location (their presumed home), weighted by the time spent at that location. | Mean, Standard Deviation |

## **Table S2.** Mean (standard deviation [SD]) of numbers of weekly passive sensing features available (i.e., at least five days of data prior to a PHQ8 score) across the 16-week study period. The maximum possible number of weekly passive sensing features is 8.

| **Passive Sensing Weekly Features** | **Mean (SD)** |
| --- | --- |
| Mean and SD of Sleep End | 7.36 (1.45) |
| Mean and SD of Sleep Start | 7.36 (1.45) |
| Mean and SD of Time in Bed | 7.36 (1.45) |
| Mean of Sleep Interruptions | 7.36 (1.45) |
| Mean and SD of Walking Rate | 7.35 (1.45) |
| Mean and SD of Step Count | 7.35 (1.45) |
| Mean of Location Entropy | 7.40 (1.42) |
| Mean and SD of Location Clusters | 7.40 (1.42) |
| Mean and SD of Distances Traveled | 7.33 (1.45) |
| Mean and SD of Time at Home | 7.36 (1.45) |

## Table **S3.** Global Passive Sensing Features. SD = Standard Deviation.

| **Weekly Summary Feature** | **Mean (SD)** |
| --- | --- |
| ***Social Engagement*** | |
| Mean Normalized Location Entropy | 0.36 (0.10) |
| Mean Log Distances Traveled | 7.91 (0.97) |
| SD Log Distances Traveled | 1.66 (0.63) |
| Mean Time at Home (hours) | 14.06 (3.46) |
| SD Time at Home (hours) | 4.55 (1.21) |
| Mean Log Number of Location Clusters | 3.26 (0.42) |
| SD Log Number of Location Clusters | 0.15 (0.05) |
| ***Activity*** | |
| Mean Step Count (per 1000 steps) | 3.55 (1.65) |
| SD Step Count (per 1000 steps) | 2.03 (0.848) |
| Mean Walking Rate (steps per second during walking events) | 1.39 (0.27) |
| SD Walking Rate (steps per minute) | 0.33 (0.08) |
| ***Sleep*** | |
| Mean Sleep Interruptions | 0.20 (0.15) |
| Mean Wake-up Time (HH:MM) | 7:34 (1:05) |
| SD Wake-up Time (HH:MM) | 01:35 (00:29) |
| Mean Bedtime (HH:MM) | 23:46 (01:14) |
| SD Bed Time (HH:MM) | 01:22 (00:24) |
| Mean Time in Bed (HH:MM) | 07:47 (1:14) |
| SD Time in Bed (HH:MM) | 1:49 (00:29) |

# **Power Calculations**

We performed a simulation study to assess power based on parameter estimates that were observed in our own data. In these simulations, we considered a range of observed model mean and variance estimates and determined the minimally detectable effect sizes (correlations) of the within- and between-person passive sensing features with the repeatedly measured depression outcome, given our study design and sample size. Specifically, we used a linear mixed effects model framework to generate data using the nlme package and the lme function in R. For each participant, we generated a passive sensing feature vector X as multivariate normal (MVN) with mean $b_{0X}$ and variance$\Sigma_{i,j}=\phi^{|i-j|}$, with ɸ=0.50 and length 8 (corresponding to eight repeated measures over the study period). To account for random variation in the mean, we used a random intercept model and allowed for the mean to vary randomly as $b_{0X}\sim MVN(0,1)$. We then computed the mean ($\bar{X}$) and within-person deviation ($\Delta_{X}$) of the passive sensing feature and used these as features to generate the depression outcome Y. Specifically, we generated Y as MVN with mean ${\mu_{Y}= b}_{0Y}{+(\beta}_{1}+b_{1})T+\beta_{2}\bar{X}+\beta_{3}\Delta_{X}$ and variance $\Sigma_{i,j}=\phi^{|i-j|}$, with autocorrelation ɸ, random intercept $b_{0Y}\sim MVN(0,1)$, random slope $b_{01}\sim MVN(0,1)$, $\beta_{1}$ = -1, and *T* as the square root of study week. Per our actual study design, we set N=131. We allowed both $\beta_{2}$ and $\beta_{3}$ to vary simultaneously to assess the strength of effect that is detectable at α=0.05 when accounting for both within and between-person effects. Finally, we converted $\beta_{2}$ and $\beta_{3}$ to correlation effect sizes to evaluate the size of the detectable effect. Our simulation study used 1000 replications and considered correlations ranging from 0.10 to 1.0 in increments of 0.10.

This simulation indicated that our study design provides > 0.80 power (α=0.05) to detect small correlations of at least *r* = 0.10 for the within-person effects and large correlations of at least *r* = 0.55 for the between-person effects. Thus, it is possible that our report may be missing some small-to-moderate between-person effects that could be clinically interesting. However, we are comfortable with this risk given that our study is already exploratory in nature. Future confirmatory studies with larger sample sizes should be used to validate and replicate our findings.

# **Model Results**

**Tables S4-S6** show model results for the passive sensing global and weekly deviation features. Each feature set (global + deviation) was considered in a separate model, adjusting for covariates. **Table S7** displays model fit indices for each model. **Figure S1** graphically depicts results related to the global mean of distances traveled.

## **Table S4.** Results for the global mean and deviation of each social engagement feature. Bolded text indicates feature was statistically significant. (SD=Standard Deviation)

| **Feature** |  | **B** | **Lower CI** | **Upper CI** | **Std Error** | **t-value** | **p-value** |
| --- | --- | --- | --- | --- | --- | --- | --- |
| **Mean Normalized Location Entropy** | **Deviation** | **-3.01** | **-5.51** | **-0.52** | **1.27** | **-2.37** | **0.018** |
|  | Global | -3.24 | -10.93 | 4.46 | 3.89 | -0.83 | 0.407 |
| **Mean Distances Traveled** | Deviation | -0.15 | -0.37 | 0.08 | 0.12 | -1.26 | 0.209 |
|  | **Global** | **-0.97** | **-1.72** | **-0.22** | **0.38** | **-2.56** | **0.012** |
| Mean Log Location Clusters | Deviation | -0.14 | -0.60 | 0.31 | 0.23 | -0.62 | 0.535 |
|  | Global | -1.33 | -2.98 | 0.31 | 0.83 | -1.61 | 0.111 |
| **Mean Time at Home** | **Deviation** | **0.05** | **0.00** | **0.10** | **0.02** | **2.12** | **0.035** |
|  | Global | 0.13 | -0.08 | 0.33 | 0.10 | 1.23 | 0.221 |
| SD Location Clusters | Deviation | 1.49 | -0.26 | 3.25 | 0.89 | 1.67 | 0.095 |
|  | Global | 5.30 | -9.42 | 20.01 | 7.43 | 0.71 | 0.478 |
| SD Distances Traveled | Deviation | -0.02 | -0.23 | 0.19 | 0.11 | -0.17 | 0.863 |
|  | Global | 0.79 | -0.34 | 1.92 | 0.57 | 1.39 | 0.167 |
| SD Time at Home | Deviation | -0.02 | -0.11 | 0.07 | 0.04 | -0.44 | 0.660 |
|  | Global | 0.15 | -0.41 | 0.72 | 0.28 | 0.54 | 0.587 |

###

## **Table S5.** Results for the global mean and deviation of each activity feature. (SD=Standard Deviation)

| **Feature** |  | **B** | **Lower CI** | **Upper CI** | **Std Error** | **t-value** | **p-value** |
| --- | --- | --- | --- | --- | --- | --- | --- |
| **Mean Total Step Count** | **Deviation** | **-0.16** | **-0.32** | **-0.01** | **0.08** | **-2.03** | **0.042** |
|  | Global | -0.41 | -0.85 | 0.03 | 0.22 | -1.84 | 0.069 |
| **Mean Walking Rate** | **Deviation** | **-1.46** | **-2.60** | **-0.32** | **0.58** | **-2.51** | **0.012** |
|  | Global | -1.95 | -4.69 | 0.78 | 1.38 | -1.41 | 0.161 |
| SD Step Count (per 1000 steps) | Deviation | -0.09 | -0.27 | 0.08 | 0.09 | -1.06 | 0.291 |
|  | Global | -0.76 | -1.59 | 0.06 | 0.42 | -1.83 | 0.069 |
| SD Walking Rate (per minute) | Deviation | -0.55 | -2.14 | 1.03 | 0.81 | -0.69 | 0.492 |
|  | Global | 5.29 | -3.54 | 14.12 | 4.46 | 1.19 | 0.238 |

## **Table S6.** Results for the global mean and deviation of each sleep feature. (SD = Standard Deviation)

| **Feature** |  | **B** | **Lower CI** | **Upper CI** | **Std Error** | **t-value** | **p-value** |
| --- | --- | --- | --- | --- | --- | --- | --- |
| Mean Wake-up Time | Deviation | 0.16 | -0.07 | 0.40 | 0.12 | 1.40 | 0.162 |
|  | Global | 0.18 | -0.47 | 0.84 | 0.33 | 0.55 | 0.585 |
| Mean Bed Time | Deviation | 0.07 | -0.20 | 0.34 | 0.14 | 0.53 | 0.597 |
|  | Global | -0.35 | -0.98 | 0.28 | 0.32 | -1.10 | 0.275 |
| Mean Sleep Duration | Deviation | 0.10 | -0.12 | 0.33 | 0.11 | 0.89 | 0.374 |
|  | Global | 0.47 | -0.13 | 1.06 | 0.30 | 1.55 | 0.123 |
| Mean Sleep Interruptions | Deviation | -0.36 | -1.49 | 0.78 | 0.58 | -0.62 | 0.536 |
|  | Global | 3.22 | -1.70 | 8.14 | 2.49 | 1.30 | 0.198 |
| **SD Wake-up Time** | Deviation | 0.14 | -0.18 | 0.46 | 0.16 | 0.89 | 0.376 |
|  | **Global** | **1.53** | **0.13** | **2.93** | **0.71** | **2.17** | **0.032** |
| SD Bed Time | Deviation | -0.01 | -0.39 | 0.38 | 0.20 | -0.04 | 0.969 |
|  | Global | 0.09 | -1.64 | 1.83 | 0.88 | 0.11 | 0.916 |
| SD Sleep Duration | Deviation | -0.02 | -0.31 | 0.27 | 0.15 | -0.14 | 0.887 |
|  | Global | 0.81 | -0.72 | 2.34 | 0.77 | 1.05 | 0.298 |

## **Table S7**. Explained variance in linear mixed effects models. Bolded items had a statistically significant between- or within-person feature in the model.

| **Passive Sensing Feature** | **Marginal R^2^*** | ***ΔR^2^***** | **Conditional R^2^***** |
| --- | --- | --- | --- |
| **Mean Normalized Location Entropy** | **0.029** | **0.005** | **0.717** |
| **Mean Distances Traveled** | **0.057** | **0.033** | **0.718** |
| Mean Log Location Clusters | 0.037 | 0.013 | 0.717 |
| **Mean Time at Home** | **0.033** | **0.009** | **0.716** |
| SD Location Clusters | 0.027 | 0.003 | 0.718 |
| SD Distances Traveled | 0.034 | 0.009 | 0.718 |
| SD Time at Home | 0.026 | 0.002 | 0.716 |
| **Mean Total Step Count** | **0.042** | **0.019** | **0.716** |
| **Mean Walking Rate** | **0.036** | **0.013** | **0.716** |
| SD Step Count (per 1000 steps) | 0.040 | 0.017 | 0.715 |
| SD Walking Rate (per minute) | 0.030 | 0.007 | 0.714 |
| Mean Wake-up Time | 0.026 | 0.002 | 0.715 |
| Mean Bed Time | 0.030 | 0.006 | 0.715 |
| Mean Sleep Duration | 0.037 | 0.013 | 0.715 |
| Mean Sleep Interruptions | 0.032 | 0.009 | 0.715 |
| **SD Wake-up Time** | **0.048** | **0.024** | **0.715** |
| SD Bed Time | 0.024 | <0.001 | 0.715 |
| SD Sleep Duration | 0.029 | 0.006 | 0.715 |

*Percent of model variance explained by fixed effects only (passive sensing features and covariates)

**Change in Marginal R^2^ after adding between- and within-person passive sensing features

***Percent of model variance explained by complete model (fixed effects and random effects)


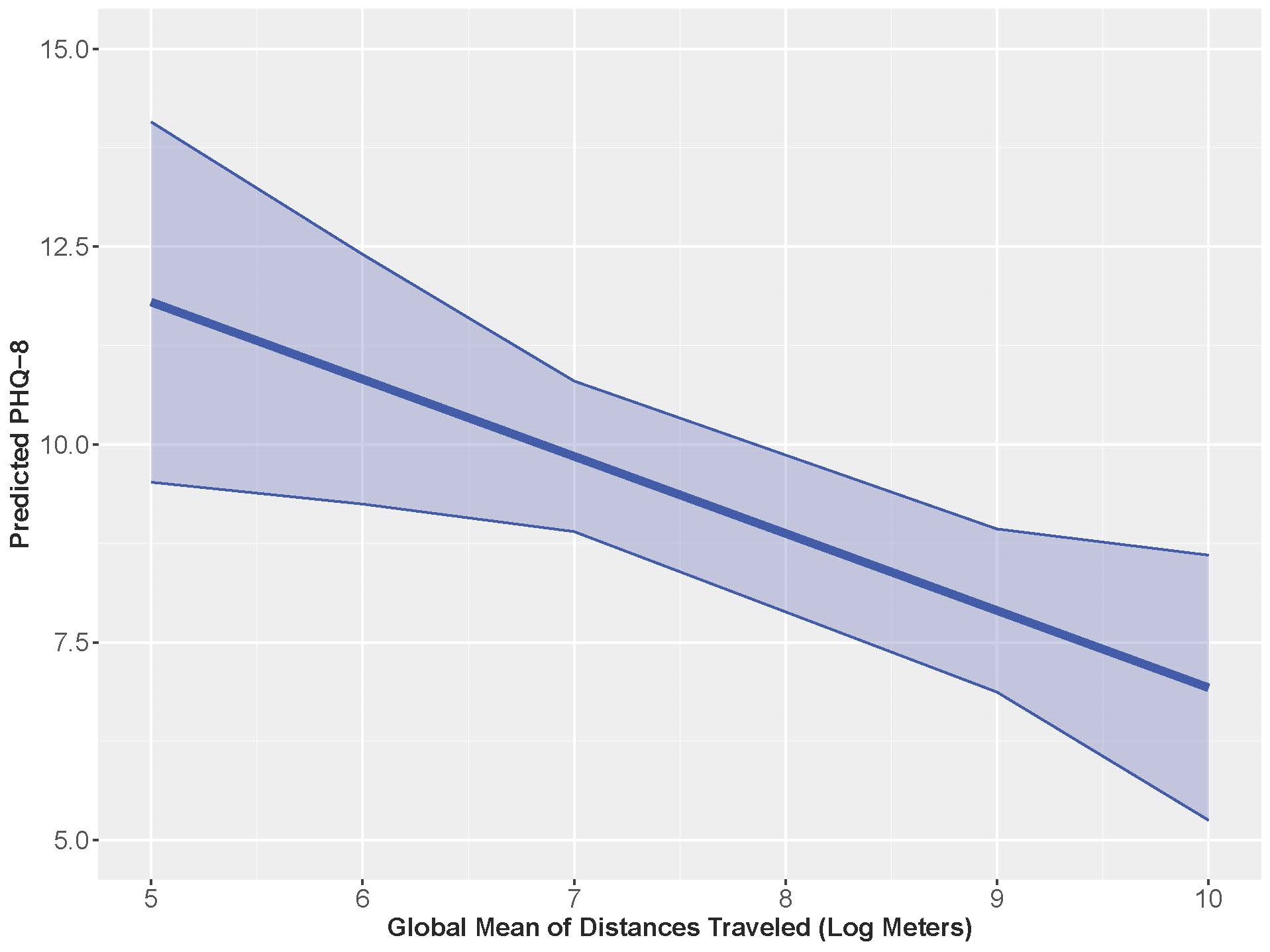


## **Figure S1.** Predicted PHQ-8 (95% Confidence Interval) for the global mean of distances traveled.

# **Illustrative Examples of Passive Sensing Features that Could Inform a Vital Sign for Depression**

**Figure S2** extends our illustration of **Figure 4** in the main text to consider how deviations of passive sensing features could be used to understand *who* has higher symptom levels. The plots in the left column correspond to ‘Participant 1’ while the plots in the right column correspond to ‘Participant 2’. The plots on the top row (‘a’) show the weekly SD of wake-up times for Participants 1 and 2, while the plots on the bottom row (‘b’) show their corresponding daily wake-up times (HH:MM). Participant 1 generally has a stable wake-up time (i.e., lower SD) and a lower PHQ-8 across the study period. In contrast, Participant 2 generally has a more variable wake-up time (i.e., higher SD) and a higher PHQ-8 across the study period.


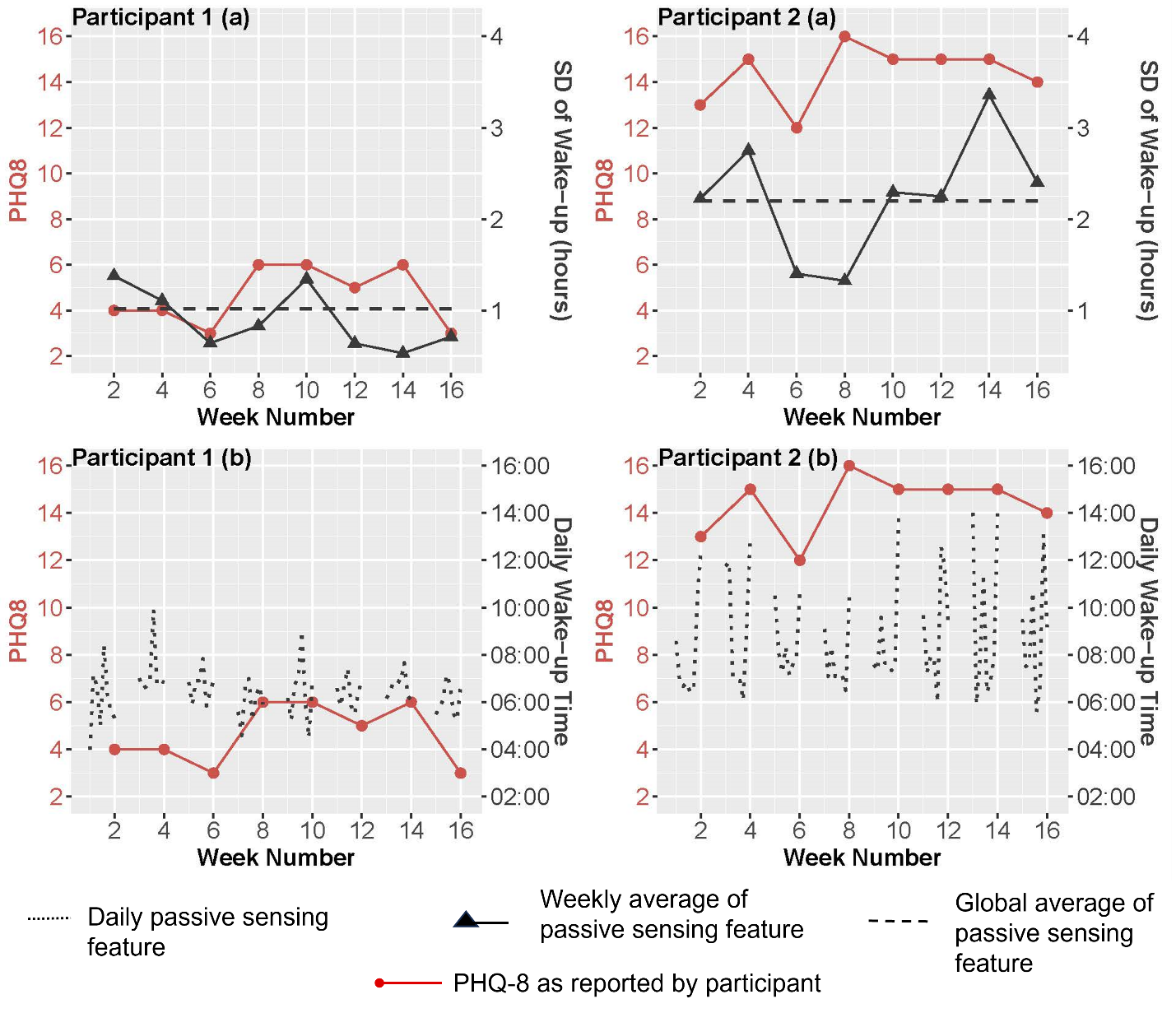


## **Figure S2**. Study participant data showing how PHQ-8 scores track with wake-up time standard deviation (SD) for two participants.
